# Malaria in Dielmo, a Senegal village: Is its elimination possible after seven years of implementation of long-lasting insecticide-treated nets?

**DOI:** 10.1371/journal.pone.0179528

**Published:** 2017-07-05

**Authors:** Amélé Nyedzie Wotodjo, Souleymane Doucoure, Jean Gaudart, Nafissatou Diagne, Fatoumata Diene Sarr, Ngor Faye, Adama Tall, Didier Raoult, Cheikh Sokhna

**Affiliations:** 1 Unité de Recherche sur les Maladies Infectieuses et Tropicales Émergentes, IRD198, UM63, CNRS7278, INSERMU1095, Aix-Marseille Université, Campus UCAD-IRD, BP, CP Dakar, Senegal; 2 Université Cheikh Anta Diop de Dakar, Faculté des Sciences et Techniques/ Laboratoire de Parasitologie, Dakar, Senegal; 3 Aix Marseille Univ, IER, INSERM, SESSTIM UMR912, Marseille, France; 4 Institut Pasteur de Dakar, Unité d'Épidémiologie des maladies infectieuses, Dakar, Senegal; 5 Institut Hospitalo Universitaire Méditerranée-Infection, Aix-Marseille Université, Unité de Recherche sur les Maladies Infectieuses et Tropicales Émergentes, IRD198, UM63, CNRS7278, INSERMU1095, Marseille, France; CNRFP, BURKINA FASO

## Abstract

**Background:**

The malaria burden has decreased significantly in recent years in Africa through the widespread use of artemisinin-based combination therapy (ACT) and long-lasting insecticide-treated nets (LLINs). However, the occurrence of malaria resurgences, the loss of immunity of exposed populations constitute among other factors, serious concerns about the future of malaria elimination efforts. This study investigated the evolution of malaria morbidity in Dielmo (Senegal) before and after the implementation of LLINs.

**Methods:**

A longitudinal study was carried out in Dielmo over eight years, from July 2007 to July 2015. In July 2008, LLINs were offered to all villagers, and in July 2011 and August 2014 the LLINs were renewed. A survey on LLINs use was done each quarter of the year. Thick smears stained with Giemsa, a rapid diagnostic test (RDT) and quantitative polymerase chain reaction (PCR) methods were performed for all cases of fever to assess malaria clinical attacks. Malaria cases were treated with ACT since June 2006.

**Results:**

Malaria morbidity has decreased significantly since the implementation of LLINs in Dielmo, together with ACT. However, malaria resurgences have occurred twice during the seven years of LLINs use. These resurgences occurred the first time during the third year after the introduction of LLINs (aIRR (adjusted incidence-rate ratio) [95%CI] = 5.90 [3.53; 9.88] p< 0.001) and a second time during the third year after the renewal of LLINs (aIRR [95%CI] = 5.60 [3.34; 9.39] p< 0.001). Sixty-nine percent (69%) of the nets tested for their long-lasting insecticidal activity remained effective after 3 years of use.

**Conclusion:**

Good management of malaria cases by the use of ACT as first-line treatment against malaria in addition to the use of LLINs has significantly reduced malaria in Dielmo and allowed to reach the phase of pre-elimination of the disease. However, the occurrence of malaria resurgences raised serious concerns about malaria elimination, which would require additional tools in this village.

## Introduction

The worldwide burden of malaria has decreased significantly during this last decade. The reduction of malaria morbidity and mortality is more pronounced in some African countries, where control strategies based on long-lasting insecticide-treated nets (LLINs) and artemisinin-based combination therapy (ACT) have been scaled up at the national level [1]. Indeed, malaria has been the subject of close attention at the international level this decade [2]. The treatment of malaria has changed from monotherapy to combination therapy, and since 2010 ACT has been used as first-line treatment in all malaria-endemic countries [3]. Depending on the country, this curative strategy was preceded or accompanied by the introduction and the use of LLINs on a large scale [4]. LLINs were attested by the World Health Organization Pesticide Evaluation Scheme (WHOPES) to retain effective pyrethroid insecticidal activity under field conditions for at least three years [5]. Consequently, the combination of these two strategies has led to an important decrease in malaria [6–10].

This dramatic decrease in malaria has stimulated the ambition of donors, policy makers and researchers to eliminate malaria [11, 12]. However, several factors could slow down or impair the efficacy of current malaria control strategies. Among these, *Anopheles* resistance to pyrethroid insecticides, which is now widespread in Africa [13, 14], may jeopardize the efficacy of LLINs. Also, the decrease of malaria incidence has induced a loss of immunity in individuals previously immunized against plasmodium parasites [15–17]. The loss of anti- *Plasmodium* immunity could increase susceptibility to clinical malaria and increase related morbidity and mortality. In addition, *Plasmodium* resistance to artemisinin which has been observed recently in East Asia [18, 19], also constitutes a threat to malaria elimination. The combination of these factors raises serious concerns about the future of malaria elimination efforts, and warning signs have been observed in endemic countries where malaria resurgences have been observed after a dramatic decrease of the disease through the large scale use of LLINs [20–25]. In Dielmo, Senegal, West Africa, malaria has significantly decreased after the implementation of universal coverage with LLINs in July 2008 and their renewal in 2011 and 2014, together with ACT. However, two malaria resurgences have occurred during the seven years of LLINs use [23, 24]. This study investigated the evolution of malaria morbidity in Dielmo in a longitudinal study over eight years: seven years of LLINs use and one year before the implementation of LLINs (the control year). The aim was to describe the significant decrease of malaria, and its resurgences, in order to provide information for malaria programs about the duration of LLINs implementation effectiveness.

## Methods

### Setting: Dielmo site

Since June 1990, a long-term research project has been conducted among the population of Dielmo, a Senegalese endemic-malaria village, in order to understand the relationship between malaria incidence, its transmission, and population immunity for different *Plasmodium* species.

The Dielmo research site has been described in detail elsewhere [26]. Briefly, the village is located in a Sudan-savannah region of central Senegal, 280 km south-east of Dakar on the marshy bank of the Nema, a small permanent stream where the persistence of anopheline breeding sites is observed year-round. Malaria transmission was continuous over the years from the beginning of the project until 2009, when transmission became seasonal. The epidemiology of malaria has changed significantly in this village, from holoendemic in 1990 to hypoendemic since 2010 [27].

### Participants and procedures

All Dielmo villagers willing to participate are involved in longitudinal follow-up, including; i) daily home-based surveillance and monitoring of all episodes of fever, including treatment and prevention of malaria attacks; and ii) repeated quarterly cross-sectional surveys to document malaria prevalence and LLIN use.

Written informed consent was obtained from all participants or from the guardians on behalf of the minors/children enrolled in the study. The study was approved by the Ministry of Health of Senegal, the assembled village population and the National Ethics Committee of Senegal.

#### Medical surveillance of fever episodes

All participating households were visited daily. The presence or absence in the village of each enrolled household member was monitored and the location of the absent member was reported. This enables computation of the number of follow-up person-days under observation for each period. In case of fever, patients were referred to the project health center, which was open 24 h/day, seven days/week, in order to be examined by a nurse. Thick smears stained with Giemsa were performed to determine the presence of the malaria parasite. Episodes of fever were attributable to *P*. *falciparum* malaria clinical attacks when parasite density was higher than an age-dependent threshold [28]. Malaria clinical attacks were treated with combination artesunate plus amodiaquine since June 2006.

From 2011 onwards, the diagnostic and treatment policy was modified to maximize efforts to limit malaria transmission, as the level of anti-malarial antibodies among Dielmo inhabitants has declined [15]. The RDT and PCR were then combined with the thick smear to improve disease diagnosis. Artesunate plus amodiaquine were systematically given to all patients with fever associated with malaria parasites detected by at least one of these diagnostic tools.

#### Cross-sectional surveys

To assess asymptomatic carriage and malaria prevalence each year, cross-sectional surveys were conducted quarterly, with two surveys during the dry season and two in the rainy season. Thick smears and RDT (since May 2011) were performed in all individuals enrolled in the Dielmo project who were present in the village during the survey.

#### Quarterly LLINs repeat cross-sectional surveys

LLINs (Permanet® 2.0) were introduced for the first time in the village in July 2008 and have been offered to all villagers. In July 2011 and August 2014, all LLINs were renewed. Simultaneously with the introduction of LLINs, repeat home-based surveys have been carried out to assess their use. Each participating household was visited quarterly during the morning by two technicians, who recorded whether the nets were hung above the bed the preceding night. They also administered a short questionnaire to household members about LLINs use. Individuals were asked if they had used nets the night preceding the visit and whether they never, always or sometimes used nets. All collected data were entered into the 4D software version 2004.5.

### Study population

In our study, we focused on person-trimester observations covering the period before and after LLINs implementation, from August 2007 to July 2015. All inhabitants of Dielmo who were enrolled in the project during this period and who had spent at least 30 days in the quarter in Dielmo were included in the study.

### Entomological studies

Since 1990, the human landing catches method (HLC) has been performed inside the concessions (two indoor HLC) and outside the concessions (two outdoor HLC) in Dielmo to assess malaria transmission. For each indoor HLC and outdoor HLC, two collectors were involved in mosquitoes catching. The households selected for the catches were unchanged during the study. The mosquito collections were performed over three consecutive nights during the first week of every month, providing an average of 12 person-nights of capture per night. The Dielmo health center provided medical surveillance for all collectors, as for the other members of the community. Entomological inoculation rates (EIR) per number of infective bites/person/night were then assessed from the monthly values for human bite rates (i.e., the number of landing mosquitoes per person) and the proportion of infected mosquitoes [29].

Thirty-two (32) random LLINs were tested after three years of use for their long-term efficacy by bioassay cones, using WHO reference tests for laboratory and field-testing of LLINs [30].

### Outcome and independent variables definition

The periods of the study were divided by the year of use of LLINs: eight years of longitudinal study, including seven years of the use of LLINs from August 2008 to July 2015 and one year before the use of LLINs (August 2007 to July 2008), used as the control year.

Malaria clinical attacks occurring among our study population were grouped together into 32 quarters over eight years (August-October, November-January, February-April and May-July of each year, previously defined). Our analysis was thus based on person-trimester observations. The outcome variable was the number of malaria attacks per person per quarter.

Rainfall was also measured each month of the study period. The rainfall was defined by the cumulative number of mm of rainfall during the previous month at the beginning of the study period in order to take into account the time interval between the occurrence of rainfall and the occurrence of malaria cases. The following variables were analyzed: i) age group, classified in six groups as follows: 0–4 years, 5–9 years, 10–14 years, 15–29 years, 30–44 years and 45 years and older; ii) rainfall; iii) sex; and iv) the year of the use of LLINs. Each variable was analyzed separately using bivariate analysis to assess the association with malaria risk. Random-effect negative binomial regression models were used to analyze clinical malaria episodes, taking into account the interdependence of successive observations in the same individuals. The days of monitoring for each person per quarter were controlled as the exposure variable. Variables that were P<0.2 in bivariate analyses were integrated in multivariate analyses [31]. Step-wise elimination of variables was performed based on the AIC (Akaike information criterion) in the model. The significance level was fixed at p = 0.05 in the final model.

Malaria morbidity was assessed by the estimation of the incidence rate. For each year of study, the malaria clinical attack incidence rate was calculated as the ratio of the number of clinical malaria attack recorded, divided by the number of person-days of follow-up during a given period. The mean monthly and yearly incidence rates were derived from the daily incidence rates based on 30.4 and 365.25 days per month and per year. The Chi-2 test for incidence rates was used to compare the incidence rate for each year.

Analyses were performed using Stata Software, version 11.0 (College Station, Texas, USA).

## Results

### Description of participants

We made 12,229 observations, corresponding to 578 individuals aged from one month to 103 years old, with a mean of 23.7 years old and a proportion of 52% of women during the study period. Among the 12,229 person-trimester observations, 491 malaria cases were noted, in whom 455 (3.7%) were related to individuals who had at least one malaria attacks per quarter between July 2007 and July 2015, and 11,774 (96.3%) observations were related to individuals who had no malaria attacks. The number of malaria attacks varied from one to three per person per quarter.

### Incidence of malaria clinical attacks over the study period

**Fig 1:**
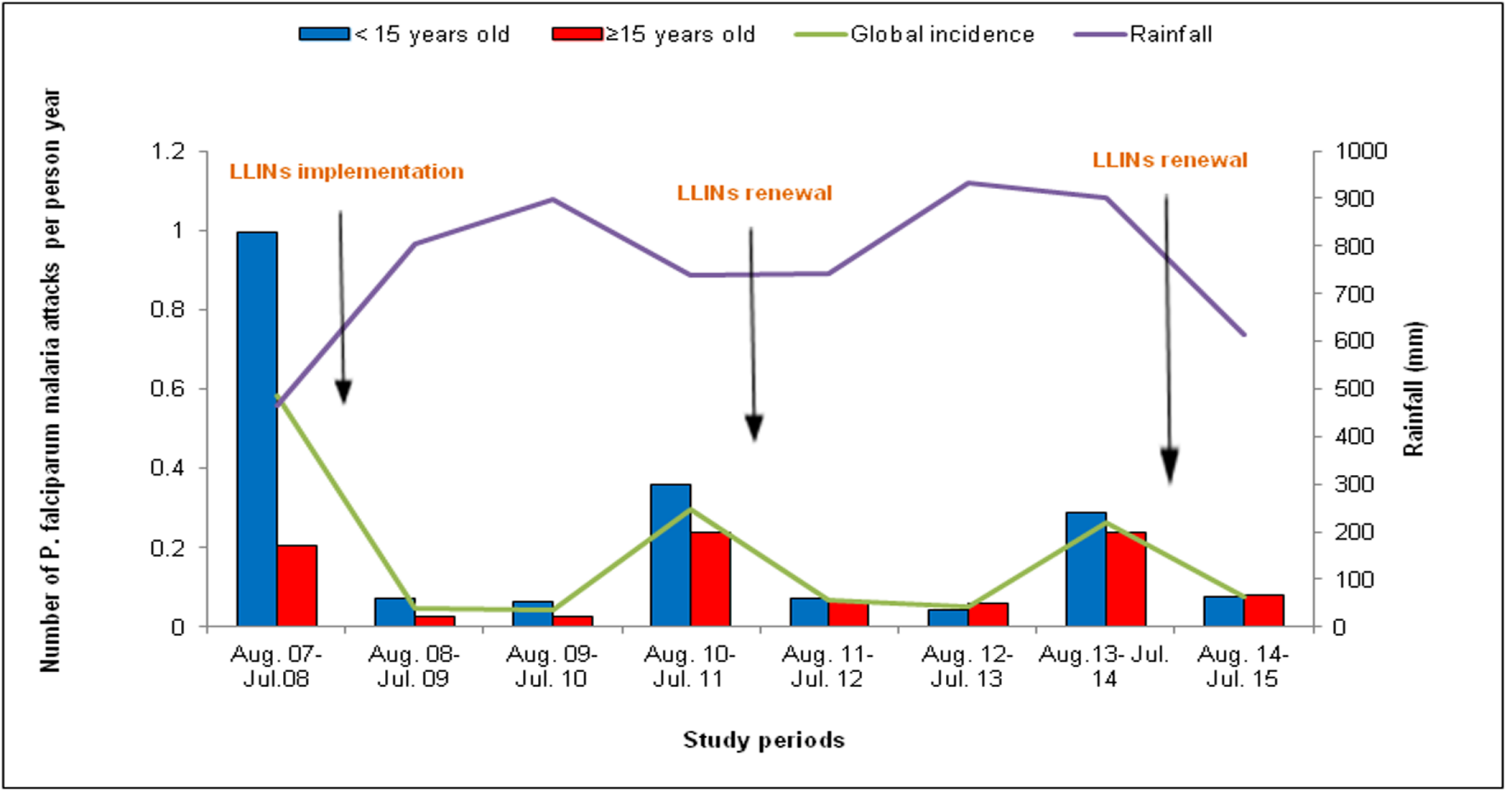
*P*. *falciparum* malaria attack incidence according to the year of Long-lasting insecticide-treated nets (LLINs) use among the population of Dielmo.

Malaria decreased dramatically after the implementation of LLINs in Dielmo. However, adults became more vulnerable; indeed, before the introduction of LLINs, only 18% of malaria cases occurred among adults, whereas since the implementation of nets, approximately 50% of cases were observed among adults (Fig 1). Fig 1 describes the evolution of malaria incidence during the entire study period among adults and children. Overall, malaria declined significantly, from 0.58 attacks per person per year before LLINs implementation to 0.05 and 0.04 attacks per person per year during the first and the second year of net use, respectively (p<0.001). Unfortunately, an upsurge of malaria was observed three years after net use, and the malaria incidence increased to 0.30 attacks per person per year. This increase was significant compared to the second year following nets implementation (p<0.001). In response to the increase in malaria, all the LLINs were renewed in July 2011. Following that, malaria consequently decreased the first and second year after the nets were renewed. The malaria incidence decreased from 0.30 attacks during the upsurge to only 0.07 and 0.05 attacks per person per year the first and the second year after the nets were renewed, respectively (p<0.001). Again, an upsurge occurred the third year after LLINs renewal, and the malaria incidence shifted to 0.26 attacks per person per year (p<0.001). Consequent to the increase in malaria, all nets were renewed for the second time in August 2014, and malaria decreased to 0.08 attacks per person per year the following year (p<0.001) (Fig 1).

### Factors associated with the risk of malaria clinical attacks

Table 1 describes both characteristics according to malaria attacks and the results of the bivariate and multivariate analyses.

**Table 1:** Socio-demographic and other characteristics according to malaria attacks and results of random-effect negative binomial regression models exploring factors associated with malaria clinical cases (n = 12,229).

		Number of observations n = 12,229 n (%)	Malaria cases		Univariate analysis		Multivariate analysis	
Characteristics	Subcategory		No n = 11,774 n(%)	Yes n = 455 n(%)	IRR (95% CI)	*P*-value	aIRR (95% CI)	*P*-value
Year of the use of LLINs								
	First year of the use of LLINs (ref)	1470 (12.02)	1454 (12.35)	16 (3.52)	1		1	
	Year before nets implementation	1404 (11.48)	1242 (10.55)	162 (35.60)	11.69 (7.11–19.20)	< 0.001	12.60 (7.65–20.74)	< 0.001
	Second year of the use of LLINs	1550 (12.68)	1536 (13.05)	14 (3.08)	0.78 (0.39–1.59)	0.50	0.77 (0.38–1.56)	0.46
	Third year of the use of LLINs	1551 (12.68)	1455 (12.36)	96 (21.10)	5.67 (3.39–9.49)	< 0.001	5.90 (3.53–9.88)	< 0.001
	Fourth year of the use of LLINs	1514 (12.38)	1494 (12.69)	20 (4.40)	1.13 (0.59–2.17)	0.70	1.17 (0.61–2.23)	0.64
	Fifth year of the use of LLINs	1567 (12.81)	1548 (13.15)	19 (4.18)	1.05 (0.55–2.02)	0.88	1.05 (0.55–2.03)	0.87
	Sixth year of the use of LLINs	1600 (13.08)	1501 (12.75)	99 (21.76)	5.56 (3.32–9.31)	< 0.001	5.60 (3.34–9.39)	< 0.001
	Seventh year of the use of LLINs	1573 (12.82)	1544 (13.11)	29 (6.37)	1.61 (0.88–2.93)	0.12	1.72 (0.94–3.14)	0.08
Age group								
	< 5years old (ref)	2028 (16.58)	1940 (16.48)	88 (19.34)	1		1	
	5–9 years old	1998 (16.34)	1888 (16.04)	110 (24.18)	0.84 (0.60–1.16)	0.28	0.97 (0.72–1.29)	0.81
	10–14 years old	1643 (13.44)	1550 (13.16)	93 (20.44)	0.74 (0.51–1.07)	0.11	0.93 (0.67–1.29)	0.66
	15–29 years old	2592 (21.20	2498 (21.22)	94 (20.66)	0.60 (0.42–0.86)	0.006	0.79 (0.57–1.10)	0.17
	30–44 years old	1771 (14.48)	1735 (14.74)	36 (7.91)	0.34 (0.21–0.54)	< 0.001	0.39 (0.25–0.61)	< 0.001
	45 years old and over	2197 (17.97)	2163 (18.37)	34 (7.47)	0.26 (0.16–0.41)	< 0.001	0.29 (0.19–0.46)	< 0.001
Sex								
	Male (ref)	5934 (48.52)	5685 (48.28)	249 (54.73)	1			
	Female	6295 (51.48)	6089 (51.72)	206 (45.27)	0.79 (0.62–1.02)	0.07		
Rainfall								
					1.0003 (0.999–1.0006)	0.06	1.0006 (1.0003–1.001)	< 0.001

IRR: Incidence rate ratio; aIRR: adjusted incidence rate ratio; LLINs: long-lasting insecticide-treated nets

The third year and sixth year after the nets implementation, as well as the year before the net implementation, were significantly associated with an increase in malaria when compared to the first year of nets implementation (incidence-rate ratio (IRR) [95%CI] = 5.67 [3.39; 9.49] p< 0.001; IRR [95%CI] = 5.56 [3.32; 9.31] p< 0.001; IRR [95%CI] = 11.69 [7.11; 19.20] p< 0.001, respectively), whereas the other years were not significantly associated with an increased malaria risk (Table 1).

Most of the bivariate analysis results were confirmed in the final multivariate model. After adjusting for potential covariates such as age, rainfall, and days of monitoring of each person per quarter, the control year (year before nets implementation), the third year and sixth year after nets implementation were significantly associated with an increase in malaria, compared with the first year of nets implementation (aIRR [95%CI] = 12.60 [7.65; 20.74] p< 0.001; aIRR [95%CI] = 5.90 [3.53; 9.88] p< 0.001; aIRR [95%CI] = 5.60 [3.34; 9.39] p< 0.001, respectively).

However, compared with the year before the implementation of LLINs when controlling for potential covariates, all the years of the use of nets were significantly associated with a decreased malaria risk (p<0.001), even if the decrease was less important for the two years of malaria upsurge compared with the other years (aIRR [95%CI] = 0.47[0.37; 0.60] and aIRR [95%CI] = 0.44[0.34; 0.57] for the third and sixth year of nets use, respectively, compared with aIRR [95%CI] = 0.08[0.05; 0.13], aIRR [95%CI] = 0.06[0.04; 0.11], aIRR [95%CI] = 0.09[0.06; 0.15], aIRR [95%CI] = 0.08[0.05; 0.14], aIRR [95%CI] = 0.14[0.09; 0.20] for the first, second, fourth, fifth and seventh year of nets use, respectively).

The malaria risk became equal among children and young adults. Indeed, compared to children less than 5 years old both in the bivariate and in the multivariate analysis, no difference in malaria risk was observed among children aged 5–9 years (aIRR [95%CI] = 0.97 [0.72; 1.29] p = 0.81), 10–14 years (aIRR [95%CI] = 0.93 [0.67; 1.29] p = 0.66) and adults aged 15–29 years (aIRR [95%CI] = 0.79 [0.57; 1.10] p = 0.17). However, older adults had fewer malaria attacks compared with children less than five years (aIRR [95%CI] = 0.39 [0.25; 0.61] p<0.001 and aIRR [95%CI] = 0.29 [0.19; 0.46] p<0.001 for adults aged 30–44 years and adults aged 45 years and over, respectively). Rainfall was significantly associated with a risk of having malaria (aIRR [95%CI] = 1.0006[1.0003–1.001], p<0.001).

### Malaria prevalence

Since the implementation of the nets, the prevalence of malaria has decreased significantly, both in children and adults. The prevalence decreased from 26% in 2007 (year before the implementation of nets) to 0.5% in 2014. During the upsurge periods, the prevalence did not increase and was 2.4% and 0.2% in 2010 and 2013, respectively.

### Entomological inoculation rate (EIR)

The EIR decreased during the period of malaria decrease, and increased during the malaria upsurges. The EIR decreased from 155.3 infective bites per person per year in 2008 to only 49.6 infective bites per person per year in 2009. However, it increased in 2010 during the upsurge period to 88.8 infective bites per person per year. In 2013, during the second malaria upsurge, the EIR increased from 7.6 infective bites per person per year in 2012 to 43.1 infective bites per person per year.

### Net use

**Fig 2:**
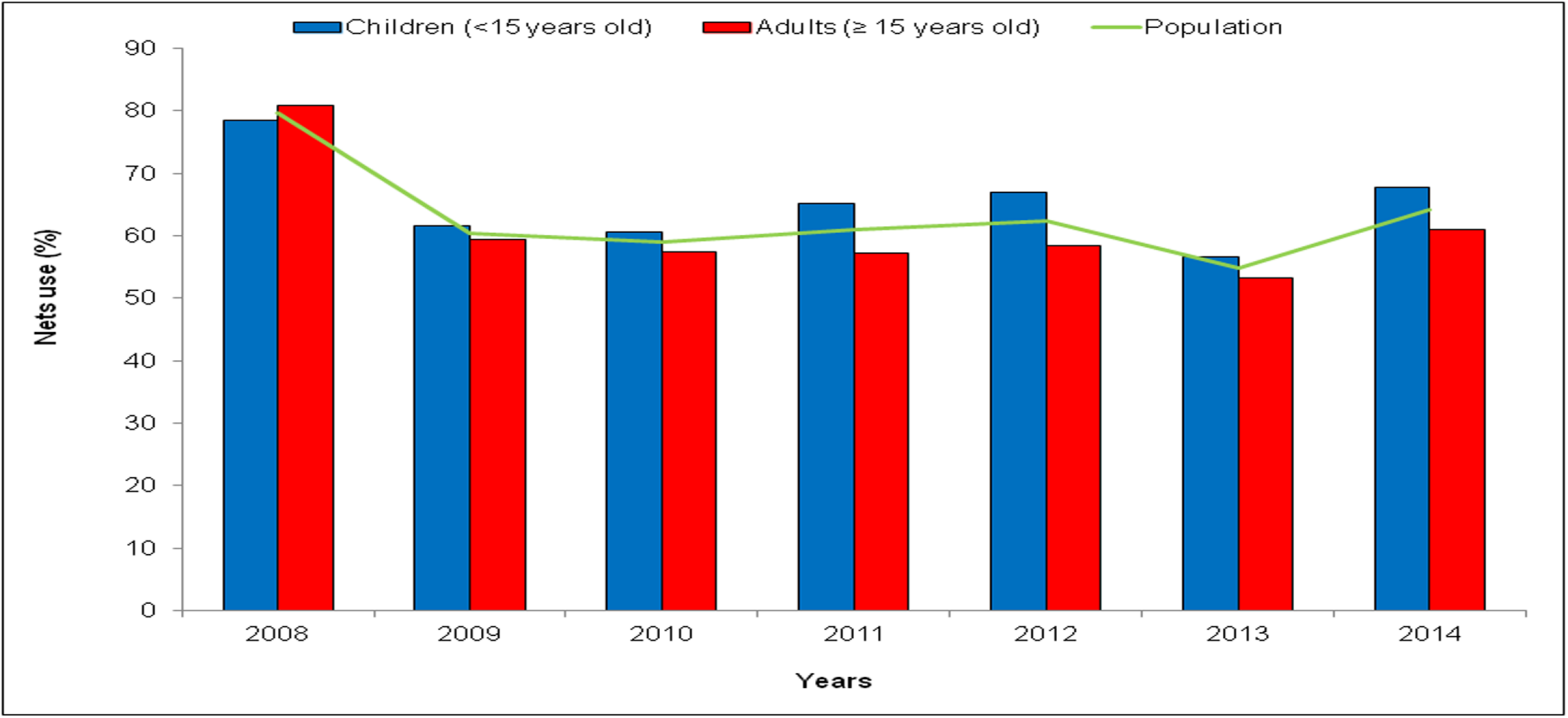
Long-lasting insecticide-treated nets (LLINs) use according to the year.

The overall use of nets was slightly greater among children than adults. The level of LLINs use was high during the first year (2008) of their implementation, with 80% of the study population sleeping under nets, but decreased to less than 70% over the following years (Fig 2). In 2009, the level of use was 61% and 59% among children and adults, respectively. During the periods of malaria upsurges, the use of nets was on average 60% and 59% during 2010–2011 and 2013–2014, respectively. The average use of LLINs from 2009 to 2014 was 63% among children and 58% among adults (χ^2^ = 32.44; p<0.001).

The selected LLINs tested for their biological efficacy remained 69% (22/32) effective for killing mosquitoes after 3 years of use.

## Discussion

Malaria has greatly decreased in some endemic countries over the last decade [1]. This dramatic decrease in the disease has stimulated the ambition of donors, policy makers and researchers to eliminate malaria [11, 12]. However much effort remains to be done for the elimination or eradication of this disease [32]. This study demonstrated the evolution of malaria clinical attacks over eight years: seven years of LLINs use and one year before the use of LLINs, where ACT is used for the first-line treatment against malaria.

The results of the study have demonstrated the efficacy of the use of LLINs to reduce malaria attacks in Dielmo, and this efficacy was reinforced by the use of ACT as the first-line treatment against clinical malaria. Indeed, the use of combination therapy as the first-line treatment against malaria in this village (amodiaquine associated with sulfadoxine-pyrimethamine, and since 2006, amodiaquine associated with artesunate) has decreased malaria morbidity approximately 2.4 times compared with the period of monotherapy treatment (data not shown). The efficacy of ACT in rapidly treating malaria attacks and reducing malaria morbidity has been observed in many studies [7, 33–35]. Some ACTs have shown their ability to reduce *Plasmodium* gametocytes and the *Plasmodium* reservoir [36, 37]. However, the current resistance of *Plasmodium* to artemisinin observed in Asia [18, 19] could constitute a threat to ACT efficacy.

The introduction of LLINs in this village was associated with a significant decrease in malaria after the first two years of net implementation. The efficacy of LLINs in significantly reducing malaria has been observed in other regions [6, 25]. However the resistance of anopheles to pyrethroid (a major insecticide used for the impregnation of LLINs) [14] and the non-regular use of LLINs, constitute an obstacle to the effectiveness of this tool [38]. The overall use of LLINs in the Dielmo population has decreased from 2009 to 2015 compared with 2008, even if the use reached 50%. LLINs are normally effective under field conditions for at least 3 years according to the manufacturers [30]. However, the resistance of anopheles to pyrethroid and the physical aspect of the nets could reduce the durability of LLINs, even if in our study most of the nets remained effective after 3 years of use.

During the study period, malaria prevalence decreased greatly and was close to zero from 2012, showing the scarcity of asymptomatic cases. Indeed, because of the probable loss of immunity [15, 23], all patient infections were symptomatic and were rapidly detected and treated. Efficient ACT treatment prevented replenishment of the parasite reservoir and maintained malaria infection at its lowest level.

This study also underlines the fragility of malaria control and elimination. Indeed, the current challenges confronting the fight against malaria were also observed in Dielmo [17, 39, 40].

One of the challenges in malaria elimination is the occurrence of the resurgence of malaria attacks. In our study area, two malaria resurgences occurred after the introduction of LLINs. The first resurgence occurred 2 years after LLINs introduction and the second resurgence occurred 2 years after LLINs renewal [23, 24]. Studies in Dielmo demonstrated that these resurgences were associated with the resistance of anopheles to pyrethroid [23], the loss of immunity against *Plasmodium* [15, 23] and the lower use of LLINs among malaria cases [24, 41]. However, there was no significant difference regarding the overall use of LLINs among the villagers during the malaria upsurge years compared with the other years of the study, except in the first year of LLINs use, when the proportion of LLINs use was the highest and reached 80%.

The results of investigation of the first resurgence showed that nets remained 60% protective against malaria despite the resistance of anopheles to pyrethroid [41]. Some studies showed that 50% of the protection of LLINs was due to it physical aspect [42]. Other studies did not find a relationship between resistance of anopheles to pyrethroid and the occurrence of malaria resurgence, and some studies did not observe the decrease of LLINs efficacy in areas of anopheles resistance to pyrethroid [43, 44]. Overall, the use of LLINs was significantly lower among persons who had malaria compared to those who did not have malaria during the first malaria resurgence, and older children and adults aged 15–29 years used their nets less than the rest of the population [41].

In July 2011, all nets were renewed, and after two years of a notable decrease in malaria, the second resurgence occurred [24]. A case control study concerning this resurgence demonstrated that LLINs remained protective against malaria, and that staying outside at night for a few hours, mainly watching television, was highly associated with malaria [24]. Electricity was introduced in this village through our project in early 2012 and allowed the population to watch television. Unfortunately, watching television outside at night exposed Dielmo inhabitants to mosquito bites [24].

These results show the challenges which currently face malaria elimination. As in Dielmo, resurgences were observed in some malaria endemic countries [20, 21, 25]. In Kenya, the resurgence of malaria prevalence was associated with decreased use of LLINs. It is one thing to possess an LLIN, it is another thing to use it; as studies show that over time, there is a decrease in the use of LLINs among populations [25, 41, 45]. Some studies have investigated low use of LLINs and have showed that some factors were significantly related to this behavior. Factors such as age (<30 years), social status, the belief in the efficacy of bed nets and their accessibility and ownership of television and radio influenced the use of mosquito nets [46–48]. A recent anthropological study has showed that the inhabitants in one locality of Benin were not using their nets, because for them, nets can cause fires, since cases of mosquito-net related burns were recorded in this locality [49].

An awareness campaign for the inhabitants of Dielmo about the beneficial effect of the use of LLINs would be a great asset and would help to prevent malaria resurgences. Anthropological studies should be conducted to understand the intentions of the Dielmo villagers about the use of LLINs. Efforts should be made to achieve 80% net use, as in 2008 in Dielmo. In addition to these efforts, increased vigilance in monitoring the occurrence of an abnormal increase of malaria cases is necessary to prevent the occurrence of a malaria epidemic in areas where malaria has notably decreased. However, it must be noted that malaria clinical cases were significantly lower during the malaria upsurge periods than during the year before the introduction of LLINs. This demonstrates that malaria resurgences, even if they are an obstacle to malaria elimination efforts, should not be compared with malaria attacks which occurred before the introduction and the use of LLINs. Although these resurgences are a threat for malaria control and elimination, they do not question the usefulness of LLINs.

Before 2008, the portion of malaria attacks among adults was only 15%, and since 2010, this portion has been approximately 50%. The malaria risk became approximately equal in all age groups, especially among children and young adults.

The increase of malaria among adults could be explained by the low use of LLINs and the decrease of immunity against *Plasmodium* [15, 23]. Adult men were often outside at night, and this behavior was shown in some studies to be a risk factor for malaria [38, 39]. This vulnerability among young adults could also be explained by the non-acquisition of immunity against malaria, since the LLINs were implemented in the village during their childhood. LLINs decrease human-vector contact and subsequently reduce the acquisition of anti-malaria immunity [50]. A study is needed in the near future to investigate this aspect by considering adults who spent part of their childhood during the use of LLINs, in order to establish the relationship between the acquisition of anti-malaria immunity among those young adults and the implementation of LLINs. These results highlight the need to take into account adults in malaria control strategies, since in the past, these strategies have been targeted on children under five years and pregnant women [51].

All these concerns underline the fragility of the important progress made in the fight against malaria. Malaria control programs should consider these concerns and integrate them into their agenda for an effective and sustainable fight against malaria, primarily in Africa.

As in Dielmo, LLINs are usually distributed freely to the population in many countries in Africa through foreign funding. As the majority of funding for the fight against malaria in African endemic countries comes from abroad, there is a question about what would happen if this funding disappears or decreases, because this funding has stagnated since 2012 [52]. In the past, the lack of resources stopped the eradication of malaria and led to malaria resurgences [53]. This could happen again if resources are limited. All governments of endemic African countries therefore need to quickly integrate malaria control programs in their national health system by unblocking a significant budget to finance malaria control activities.

Malaria elimination is possible in Dielmo if these challenges can be controlled. However, the persistence of malaria in areas and villages around Dielmo constitutes another challenge [54]. Dielmo is a type of experimental research village for malaria, and the results obtained in this village should help decision makers in their strategies for the control and elimination of malaria. The elimination of malaria, in view of the results presented here, seems possible in Dielmo, but much more is needed to achieve this. The current means used in the fight against malaria make possible a control and pre-elimination of the disease as shown by the results obtained in the village of Dielmo. Proper management of malaria cases with ACT in addition to the use of LLINs and good monitoring of the population have dramatically reduced malaria in Dielmo, achieving the control and pre-elimination phase of the disease. However, maintaining this pre-elimination phase or eliminating malaria would require more tools, such as mass drug administration, indoor residual spraying and seasonal malaria chemoprevention.
